# Contextual Fear Conditioning Alter Microglia Number and Morphology in the Rat Dorsal Hippocampus

**DOI:** 10.3389/fncel.2019.00214

**Published:** 2019-05-14

**Authors:** Nicholas Chaaya, Angela Jacques, Arnauld Belmer, Kate Beecher, Syed A. Ali, Fatemeh Chehrehasa, Andrew R. Battle, Luke R. Johnson, Selena E. Bartlett

**Affiliations:** ^1^School of Clinical Sciences, Queensland University of Technology, Brisbane, QLD, Australia; ^2^Institute of Health and Biomedical Innovation, Translational Research Institute, Queensland University of Technology, Brisbane, QLD, Australia; ^3^School of Biomedical Sciences, Queensland University of Technology, Brisbane, QLD, Australia; ^4^Diamantina Institute, The University of Queensland, Brisbane, QLD, Australia; ^5^School of Psychology and Counselling, Queensland University of Technology, Brisbane, QLD, Australia; ^6^Center for the Study of Traumatic Stress, Department of Psychiatry, Uniformed Services University School of Medicine, Bethesda, MD, United States

**Keywords:** contextual fear conditioning, microglia, BDNF, hippocampus and amygdala, dentate gyrus

## Abstract

Contextual fear conditioning is a Pavlovian conditioning paradigm capable of rapidly creating fear memories to contexts, such as rooms or chambers. Contextual fear conditioning protocols have long been utilized to evaluate how fear memories are consolidated, maintained, expressed, recalled, and extinguished within the brain. These studies have identified the lateral portion of the amygdala and the dorsal portion of the hippocampus as essential for contextual fear memory consolidation. The current study was designed to evaluate how two different contextual fear memories alter amygdala and hippocampus microglia, brain derived neurotrophic factor (BDNF), and phosphorylated cyclic-AMP response element binding (pCREB). We find rats provided with standard contextual fear conditioning to have more microglia and more cells expressing BDNF in the dentate gyrus as compared to a context only control group. Additionally, standard contextual fear conditioning altered microglia morphology to become amoeboid in shape – a common response to central nervous system insult, such as traumatic brain injury, infection, ischemia, and more. The unpaired fear conditioning procedure (whereby non-reinforced and non-overlapping auditory tones were provided at random intervals during conditioning), despite producing equivalent levels of fear as the standard procedure, did not alter microglia, BDNF or pCREB number in any dorsal hippocampus or lateral amygdala brain regions. Despite this, the unpaired fear conditioning protocol produced some alterations in microglia morphology, but less compared to rats provided with standard contextual fear conditioning. Results from this study demonstrate that contextual fear conditioning is capable of producing large alterations to dentate gyrus plasticity and microglia, whereas unpaired fear conditioning only produces minor changes to microglia morphology. These data show, for the first time, that Pavlovian fear conditioning protocols can induce similar responses as trauma, infection or other insults within the central nervous system.

## Introduction

Contextual fear conditioning (CFC) is a Pavlovian conditioning protocol whereby an animal, typically a rodent, is placed into a context (conditioned stimulus; CS) and provided with noxious stimuli (unconditioned stimulus; US) (Foa et al., 1992; Rothbaum and Davis, 2003; Fanselow, 2010; LeDoux, 2014; Chaaya et al., 2018). CFC, along with similar fear conditioning protocols are utilized to replicate the behavioral events that lead to the development of fear-based disorders, namely, post-traumatic stress disorder (PTSD) (Foa et al., 1992; Rothbaum and Davis, 2003; Fanselow, 2010; Maren, 2011; LeDoux, 2014; Chaaya et al., 2018). Utilizing these conditioning protocols, researchers have identified various essential brain regions, circuits and molecules involved in the consolidation, maintenance, expression, recall, and extinction of fear (Fanselow, 2010; Maren, 2011; LeDoux, 2014). During CFC, the basolateral amygdala complex (BLC) and hippocampus; or more specifically, the lateral amygdala (LA) and dorsal hippocampus (DH), have been identified as two brain regions critical for its consolidation (Chaaya et al., 2018). Numerous investigations, largely directed by Fanselow, have demonstrated an inability for contextual fear memories to be consolidated when these regions are inhibited or abolished (Maren and Fanselow, 1997; Maren et al., 1997; Anagnostaras et al., 1999; Fanselow, 2010). Additional investigations exploring cellular and molecular alterations following learning in these regions have also identified a critical role for various plasticity and activity related proteins and immediate early genes (IEGs) in the LA and DH following CFC (Impey et al., 1998; Sananbenesi et al., 2002; Perez-Villalba et al., 2008; Barot et al., 2009; Wilson and Murphy, 2009; Pignataro et al., 2013; Besnard et al., 2014; Zelikowsky et al., 2014; Zheng et al., 2015; Choi et al., 2016; Chaaya et al., 2019). The current study expands on these studies and our previous work (Chaaya et al., 2019) which demonstrated how minor changes to the context during CFC alter LA activity. This study explores a variety of cellular and molecular alterations in LA as well as the DH.

The unpaired fear conditioning (UFC) protocol is an alternate Pavlovian conditioning procedure capable of robustly producing contextual fear memories (Trifilieff et al., 2007; Bergstrom et al., 2011, 2012). Despite this, UFC protocols have traditionally been utilized as controls for cued fear learning (e.g., auditory fear conditioning; AFC) procedures (McKernan and Shinnick-Gallagher, 1997; Rogan et al., 1997; Majak and Pitkänen, 2003; Radley et al., 2006; Bergstrom et al., 2011, 2012). While the CS (e.g., tone) and US (e.g., foot-shock) are explicitly paired in cued fear conditioning, they are presented at random non-overlapping times during UFC. As the noxious and neutral stimuli (foot-shock and tone) are non-overlapping during UFC, this led to the hypothesis that the amygdala would not be activated, as no explicit CS-US associative memory was formed (McKernan and Shinnick-Gallagher, 1997; Rogan et al., 1997; Majak and Pitkänen, 2003; Radley et al., 2006; Bergstrom et al., 2011, 2012). However, UFC robustly produces contextual fear memories, and, as noted above, contextual fear memories activate both LA and DH. Therefore, the ability for fear memories to context to be formed following UFC suggest the LA, DH, or other related brain region are similarly recruited. Indeed, this has been demonstrated previously in LA by Trifilieff and colleagues [quantified phosphorylated mitogen-activated protein kinase; pMAPK (Trifilieff et al., 2007)], and more recently by our lab (quantified IEGs Chaaya et al., 2019). The current study aims to expand on this research by exploring additional molecular and cellular alternations in both DH and LA following CFC and UFC.

Microglia are functionally and anatomical distinct central nervous system (CNS) cells that possess macrophage-like function (Walker et al., 2014). They are crucially involved in responding to infection, trauma, ischemia and other insults of the brain, and participate in maintaining neuronal integrity (Kettenmann et al., 2011; Pósfai et al., 2018). Briefly, microglia respond to insult in two main ways: they increase in number in the affected area and their morphology (cell body size and extension number/size) alter (Kettenmann et al., 2011; Calcia et al., 2016). Resting (ramified) microglia in a healthy CNS system have a small cell body and long, thin extensions, with many processes (Kettenmann et al., 2011; Calcia et al., 2016; Dwyer and Ross, 2016). In this ramified stage microglia search for signals of insult (Kettenmann et al., 2011; Walker et al., 2014; Dwyer and Ross, 2016). Upon detection of harmful stimuli, microglia number increase in the affected area, and morphology alter to become amoeboid, with most notable changes being an increase in cell body size, and reduced branching, and number of extensions (Kettenmann et al., 2011; Walker et al., 2014; Dwyer and Ross, 2016). Furthermore, in this state, microglia release a number of factors and compounds, one such being brain derive neurotrophic factor (BDNF), an important neurotrophin involved in neuronal survival and differentiation (Ferrini and De Koninck, 2013; Pósfai et al., 2018). Recent research has begun to identify how microglia respond to psychosocial stressors, with various studies (extensively reviewed by Calcia et al., 2016) showing alterations corresponding to that of an injured or insulted brain. To our knowledge, one study has identified such alterations following chemically induced fear (Vollmer et al., 2016). However, no research has directly examined how Pavlovian fear conditioning protocols alter microglia number and morphology. The current study, is therefore designed to examine how CFC and UFC result in alterations in microglia number and morphology. Furthermore, we investigate how these protocols alter BDNF expression (which can be released by microglia), as well as phosphorylated cyclic-AMP response element binding (pCREB) expression (plasticity marker that can be activated by BDNF) (Minichiello, 2009; Ferrini and De Koninck, 2013). Both pCREB and BDNF expression have been demonstrated to be involved in fear memory formation, making them good candidate proteins for the investigation of differential fear memory consolidation (Impey et al., 1998; Hall et al., 2000; Liu et al., 2004; Bekinschtein et al., 2007; Mamiya et al., 2009; Mizuno et al., 2012).

The objectives of the current study were to explore how two different Pavlovian fear conditioning protocols, capable of creating contextual fear memories, alter LA and DH microglia (identified by labeling for ionized calcium binding adaptor molecule 1; IBA-1) number and morphology, BDNF number and pCREB number. The current study provides the first insights into how these Pavlovian fear conditioning protocols alter microglia number and morphology. We find that microglia number and BDNF expression increase in the dentate gyrus (DG) subregions of the DH, following CFC, as compared to a context only (CO) control group. Further investigations show that CFC alter microglia morphology to become amoeboid (responding to insult). Interestingly, we show that UFC does not lead to a change in BDNF or microglia number. Despite this, investigations of microglia morphology in UFC suggest they also appear to have some characteristics of amoeboid microglia; signifying they also responded to insult.

## Materials and Methods

### Animals

Animals were experimentally naïve adult male Sprague Dawley Rats (Animal Resources Centre, WA, Australia). Data reported here was gathered from rats that make part of a larger dataset. Rats weighed 176–200 g at arrival and were housed, two per cage, by the University of Queensland Biological Resources (UQBR) facility on a 12-h light/dark cycle. Food and water was provided *ad libitum*. All behavioral procedures were approved by the University of Queensland (Ethics approval no. 023/17) and Queensland University of Technology (QUT approval no. 1700000295) animal ethics unit. All procedures complied with the Queensland Government Animal Research Act 2001, associated Animal Care and Protection Regulation (2002 and 2008), as well as the Australian Code for the Care of Animals for Scientific Purposes, 8th Edition (National Health and Medical Research Council, 2013) policies and regulations of animal experimentation and other ethical matters. Upon arrival, rats were acclimatized to the UQBR Facility for 8 days, handled by the experimenter for 9 days, habituated to the fear conditioning context for 1 day, and then, 24 h later, fear conditioned (now weighing 326.56 ± 2.8 g on the fear conditioning day) as explained previously (Chaaya et al., 2019). There were two experimental (Contextual Fear Conditioned; CFC *n* = 18, Unpaired Fear Conditioned; UFC *n* = 18) groups and one control (Context Only; CO *n* = 18) group. Rats were divided into anatomical (*n* = 12 per group) and behavioral (*n* = 6 per group) subgroups following experimental procedures.

### Apparatus

All procedures occurred in one of two Plexiglas conditioning chambers (Coulbourn Instruments, Lehigh Valley, Pennsylvania, United States). A single house light (2–3 lux) dimly illuminated both chambers (context A and B). Chambers contained an infrared camera, were equipped with a speaker and sound insulated (background dB = 55). Context A contained a metal grid floor which connected to an electric shock generator. This context contained no decorations, and was cleaned with ethanol (EtOH) 80% following the presentation of each rat. Alternatively, context B was fitted with a flat floor that was lightly covered with bedding. The walls were colored, and alterations were made to the roof which altered its physical dimensions. Following the presentation of each rat, orange scented hand soap was used to clean context B. The bedding was also replaced.

### Procedures and Design

**FIGURE 1:**
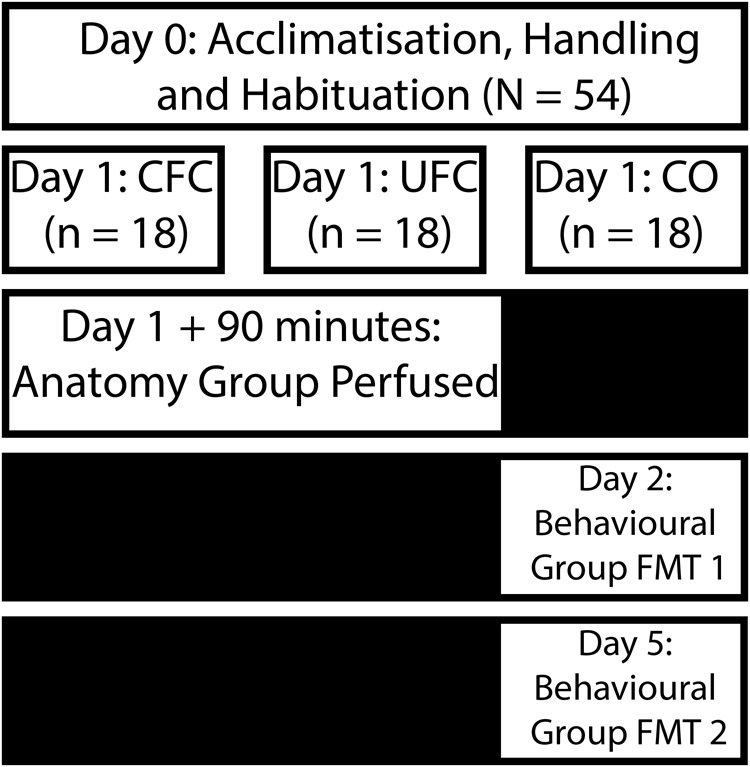
Experimental design for behavioral training. Following an acclimatization, handling and habituation period, rats were divided into three distinct behavioral groups. Rats in the CFC group were placed into a chamber and provided with five non-overlapping and random electric shocks to the foot. Rats in the UFC were placed in the same chamber and provided with the same foot-shocks. However, five non-overlapping and random auditory tones were also presented during the fear conditioning procedure. Rats in the CO control group were placed in the same chamber and provided with no further stimuli. Following conditioning, rats in all three groups were separated into an anatomy (perfused 90 min post-conditioning) and behavioral group (provided with two fear memory test 24 h after conditioning, and 4 days after conditioning). CFC, contextual fear conditioning; UFC, unpaired fear conditioning; CO, context only.

Figure 1 briefly outlines behavioral procedures. These procedures are explained in detail below and have been outlined previously (Chaaya et al., 2019).

#### Acclimatization, Habituation, and Fear Conditioning

Prior to behavioral procedures, rats in all conditions were acclimatized to the vivarium for 8 days. Rats were handled for 9 days by the experimenter, and then each placed in context A for 30 min on the 10th day. After 24 h, rats in the CFC and UFC group were placed into context A for fear conditioning. Rats were permitted 180 s to explore the context before receiving any stimuli. Rats in the CFC group were then presented with five non-overlapping and random electric shocks to the foot (1.0 mA, 0.50 s). Whilst in receiving these foot-shocks in context A, rats in the UFC group also received five presentations of auditory tones (5 kHz, 75 dB, 20 s). These auditory tones did not overlap with each other, or with the foot-shocks. Following presentation of the final stimulus, rats remained in the context for 60 s. The experimenter then removed the rats, and returned them to their home-cage. In total, fear conditioning procedures were 660 s long for rats in the CFC group, and 880 s long for rats in the UFC (extra time was required to account for the addition of auditory tones). Rats in the CO control group were placed into context A without any added stimuli, and left to explore for 660 s.

#### Following Fear Conditioning

##### Behavioral subgroup

The behavioral subgroup of rats (*n* = 6 per group) had freezing behavior manually scored during training (fear conditioning) and testing (fear memory test: FMT). As per previous investigations (Phillips and LeDoux, 1992, 1994; Quirk et al., 1997; Radley et al., 2006; Bergstrom et al., 2012; Bergstrom and Johnson, 2014), all scoring occurred in 20 second blocks. During training a progressive measure of fear was obtained by scoring freezing behavior before (baseline), during (cue 1–5), and after fear conditioning (final). Following training, rats were returned to their home-cages, and kept there for 24 h. Rats were then placed back into context A, and freezing behavior was scored for a 10 min FMT to context (no foot-shocks or auditory tones provided). Freezing behavior was scored during the final 20 s of every minute that rats were undergoing their FMT to context. Rats were returned to their home-cages for 72 h, until which a FMT to tone was conducted. During the FMT to tone, rats were placed in context B for 10 min and presented with auditory tones (5 kHz, 75 dB, 20 s) at the end of each minute. Freezing behavior was scored during the 20 s tone presentation periods. Providing the FMT to context prior to the FMT to tone was required to ensure no extinction learning to context occurred. Fear extinction (reduction in fear response) occurs when rodents are re-exposed to the fear conditioning context without the foot-shock (Bouton, 1993; Bouton, 2004). Additionally, generalization of fear is a well-documented phenomenon whereby fear memory generalizes (spreads) from one context to another similar, but altered, context (Wiltgen and Silva, 2007; Xu and Südhof, 2013). Contextual fear extinction may have occurred during the FMT to tone if fear to context A (where fear conditioning occurs) generalized to context B (where FMT to tone occurs). Therefore, to ensure fear to context was accurately and fully measured, the FMT to context was provided first. Importantly, fear to tone could not be affected by the FMT to context, as the tone is in no way presented during this FMT. This method coincides with previous investigations (Phillips and LeDoux, 1994; Bergstrom et al., 2012), whereby fear to tone remained robust (96% in Bergstrom et al., 2012) when tested 3 days after a FMT to context.

##### Scoring of freezing

Freezing behavior was defined as the inhibition, absence or suppression of movement, besides from that required for autonomic nervous system functioning (Fanselow, 1980). Head scanning and sleeping were not included as freezing. Heavy breathing, minimal movement and other movements required for normal respiration and autonomic function were considered as freezing behavior. During training, freezing behavior was scored in the CFC and UFC groups during the final 20 s of the first minute, the final 20 s of the last minute, and the 20 s prior to each foot-shock. Freezing behavior was not scored prior to auditory tone presentation in the UFC, as the development of contextual fear memories are of interest here. For rats in the CO control group, freezing behavior was scored at identical time points as that of the CFC group (as their trials had identical durations). During testing, freezing behavior was scored in the final 20 s of every minute (Bergstrom et al., 2011, 2012).

##### Anatomical group

Following completion of fear conditioning, rats in the anatomical group (*n* = 12 per group) were removed from the conditioning context and immediately returned to their home cages. Ninety minutes following, rats were anesthetized and sacrificed via perfusion for fluorescent labeling of pCREB, BDNF and IBA-1. This time point was selected to accurately identify changes in pCREB and BDNF expression, which have been shown to increase between 30 min and 120 min following learning or other behavioral and anatomical manipulations (Shiromani et al., 1995; Hall et al., 2000; Stanciu et al., 2001; Rasmusson et al., 2002; Barrientos et al., 2004; Lee et al., 2004; Izumi et al., 2008; Lubin et al., 2008; Takei et al., 2011; Mizuno et al., 2012).

### pCREB, BDNF, and IBA-1 Immunohistochemistry

#### Tissue Preparation

To anaesthetize rats, intraperitoneal (i.p.) injections of Ketamine/Xylazine (100 mg/kg, 10 mg/kg) were administered. Following anaesthetization, rats were transcardially perfused with ice-cold saline (200 mL per rat) followed by 4% paraformaldehyde/0.1 M phosphate buffer (PB; pH of 7.4; 400 mL per rat) via the ascending aorta. Subsequently, brains were removed and stored at 4°C in the 4% paraformaldehyde fixative for 24 h. Following, brains were stored in phosphate buffered saline (PBS)/0.02% azide for a minimum of 3 days, at which point free-floating sequential coronal brain sections were obtained. These sections, sliced on a vibratome (M11000; Pelco easiSlicer, Ted Pella Inc., CA, United States) at 40 μm per section, contained the lateral amygdala and the hippocampus. Sections were stored at 4°C in PBS/0.02% azide until immunohistochemistry commenced. Immunohistochemistry was conducted on right hemisphere sections.

#### Immunohistochemistry

Brain sections were removed from PBS/0.02% azide and washed thoroughly with PBS. Optimization of each antibody required altered protocols. These alterations are outlined below. First, all sections were post-fixed for an additional 5 min with the 4% paraformaldehyde fixative used for perfusion. Sections were then thoroughly washed with PBS. Sections labeled for BDNF and IBA-1 (but not pCREB) were incubated in 3% H_2_O_2_/10% Methanol in PBS for 5 min. Once washed in PBS, all sections were permeabilised with 1% Triton/0.1% Tween 20 in PBS for 1 h, and then washed in PBS again. Labeling for BDNF required antigen retrieval; whereby sections were incubated in Citrate Buffer (10 mM Sodium Citrate, 0.05% Tween 20, pH 6.0) for 5 min (80°C). Once sections returned to room temperature, they were washed in PBS. All sections were then blocked with 0.3% Triton/0.05% Tween 20/2% normal horse serum (NHS) in PBS for 1 h. Blocking solution was removed, and sections were incubated in their respective primary (either pCREB, BDNF, or IBA-1) antibody diluted in the blocking solution for 24 h. Sections labeled for pCREB were incubated in anti-phospho-CREB (Ser133) rabbit polyclonal antibody (1:500; Merck Millipore, HE, DEU). Sections labeled for BDNF were incubated in anti-BDNF [EPR1292] (ab108319) rabbit monoclonal antibody (1:500; Abcam, VIC, Australia). Sections labeled for IBA-1 were incubated in anti-IBA1 (ab5076) goat polyclonal antibody (1:500; Abcam, VIC, Australia). Following incubation in primary antibody, all sections were washed with blocking solution. Sections labeled for pCREB and BDNF were immediately incubated in a pre-absorbed goat anti-rabbit IgG H&L (Alexa Fluor 594) secondary antibody (1:500; Abcam, VIC, Australia) in blocking solution, whereas sections labeled for IBA-1 were immediately incubated in a cross-absorbed donkey anti-sheep IgG H&L (Alexa Fluor 594) secondary antibody (1:500; Thermo Fisher Scientific, VIC, Australia). Brain sections were then washed in blocking solution and then PBS. Following, sections were incubated in 4′,6-diamidino-2-phenylindole (DAPI) diluted in PBS for 5 min (1:1000; D1306 Thermo Fisher Scientific, VIC, Australia), washed a final time, and then mounted on silane coated slides. Mounted sections were immediately cover-slipped using ProLong Gold antifade reagent (Invitrogen, CA, United States), left to dry and stored at 4°C.

#### Image Acquisition

Cover-slipped brain sections were scanned using a Nikon/Spectral Spinning Disk Confocal Microscope (Nikon Instruments Inc., NY, United States) to take 20× magnified tile-scan mosaics of the amygdala and hippocampus. Scan settings are as follows: *x* = 14, *y* = 10 consecutive fields (horizontal acquisition pattern) with 10% overlap and 7 z-stacks with 4 μm step-size. Laser channels were 405 nm for DAPI (20 ms exposure time) and 561 nm (high) for pCREB, BDNF, and IBA-1 (300 ms exposure). Individual scans (each z-stack and wavelength/channel as a separate image) were saved as separate .tiff files, and manually merged using ImageJ (Schindelin et al., 2012). Merged z-stacks and channels were then stitched (Preibisch et al., 2009) in ImageJ. The Olympus FV3000 Confocal Laser Scanning Microscope (Olympus Australia Pty Ltd., VIC, Australia) was used to take 40× magnified (1.5× zoom) scans (*x* = 212 μm, *y* = 212 μm) with 30 z-stacks of 0.50 μm thickness (*z* = 15 μm). These scans were only conducted on IBA-1 sections to allow for the tracing of these cells. Only brain subregions that were identified to have significant differences in pCREB, BDNF and IBA-1 number (as identified with initial Spinning Disk Confocal scans) were scanned with the FV3000.

#### Amygdala and Hippocampus Subregion Identification

All brain sections contained the LA and its three subregions: dorsolateral portion of the lateral amygdala (LaDL), ventromedial portion of the lateral amygdala (LaVM), and ventrolateral portion of the lateral amygdala (LaVL), as well as the dorsal hippocampus (DH) and its three subregions: dentate gyrus (DG), CA1 and CA3. The CA2 was excluded from analysis as it is significantly smaller than the other hippocampal subregions, and is difficult to accurately outline (Caruana et al., 2012). Stereotaxic alignment was utilized to accurately analyze pCREB, BDNF and IBA-1 expression in the same rostral-caudal location of each rat brain. Briefly, the lateral ventricle (LV) – a rapidly changing anatomical landmark – becomes present at Bregma coordinate -3.32 mm, and consistently grows towards more caudal locations (Paxinos and Watson, 2006). The LV becomes easily identifiable at Bregma coordinate -3.36 mm (depicted as a tear-drop size; Paxinos and Watson, 2006), allowing for accurate identification and alignment. Following identification of Bregma coordinate -3.36 mm, preceding sections can be identified by counting back. Identification of Bregma coordinate -3.36 mm allowed for all rat brains to be aligned at the same rostral-caudal location. To provide a larger representation of the LA and DH, two sections per rat (equal distances apart) were labeled with each antibody. Anatomical landmarks, such as the external capsule (ec), rhinal fissure (RF), central amygdala (CeA), dorsal endopiriform nucleus (DEn), optic tract (opt), and stria terminalis (st) were used to identify the three LA subregions (see Figure 3A, 4A, 5A). The hippocampal subregions were more easily identified by the clear structural alterations of each subregion (see Figure 3B–D, 4B–D, 5B–D).

### Neuron/Microglia Quantification

Neurons and Microglia were automatically counted and tagged using the spot detection option in IMARIS (IMARIS 9.1.2, Bitplane, Zurich, Switzerland). An average diameter was obtained for cells labeled with pCREB, BDNF and IBA-1. Filter intensity was determined by the experimenter, and kept constant across experimental groups. Cell diameter and filter intensity were altered, if necessary, depending on amygdala or hippocampus subregion, but remained constant across experimental groups for each subregion. Representative areas of LA subregions (*x* = 266.66 μm, *y* = 266.66 μm) were selected and the number of cells and microglia quantified (see Figure 3A, 4A, 5A). Due to the relatively large size of the DH and its subregions, three areas (*x* = 333.33 μm, *y* = 333.33 μm) were counted from each DH subregion (see Figure 3B–D, 4B–D, 5B–D). Tracing of microglia was dependent upon pCREB, BDNF and IBA-1 quantification. In identified brain subregions, a maximum of three microglia cells per section were traced using Neurolucida 360 (Neurolucida 360, MBF Bioscience, VT, United States). The average length of microglia extensions, number of trees or ends for these extensions, the cell body volume and complexity of the microglia branching (measure of ramification state) were quantified from these traces. The following formula was utilized to determine the complexity of microglia branching (sum of the terminal orders + number of terminals) ^∗^ (total process length/number of primary branches) (Pillai et al., 2012).

### Data Analysis

The results section is separated into three parts: pCREB expression, BDNF expression and IBA-1 expression. Within each part, differences as a function of conditioning (CFC, UFC, and CO control) across all LA and DH subregions are explored. For this reason, analyses of variances (ANOVAs) were utilized to compare differences between behavioral groups within each subregion (e.g., differences in pCREB expressing neurons between behavioral groups in the LaDL). Prior to analysis, normality and homogeneity of variance were tested for. Assumptions of normality and homogeneity of variance were confirmed in the majority of cases. To control for possible type I errors arising from these breaches, a Bonferroni adjustment (Perneger, 1998) was utilized in all follow-up *post hoc* tests. The Bonferroni adjustment also controlled for multiple comparisons that were conducted in these analyses. Therefore, all analyses to anatomical data were conducted with one-way ANOVAs, followed by Bonferroni corrected *post hoc* tests. All values in the text and graphs are expressed as the mean ± standard error of the mean. *P*-values at or below 0.05 are considered statistically significant. All major statistical analyses, outlier analyses and graph generation were conducted using GraphPad Prism v7 software (GraphPad, CA, United States). Asterisks are used to denote levels of statistical significance within all graphs (^∗^*p* ≤ 0.05; ^∗∗^*p* ≤ 0.01; ^∗∗∗^*p* ≤ 0.001; ^∗∗∗∗^*p* ≤ 0.0001). Behavioral data is previously reported in MS1 (Chaaya et al., 2019), and therefore summarized briefly at the beginning of the results section.

#### Excluded Cases

Statistical outliers or significantly damaged brain tissue (occurring from perfusion, labeling or cover-slipping process) were excluded from analyses. Statistical outliers were identified via the ROUT method (GraphPad Prism), with the maximum false discovery rate set to 1%. The ROUT method is capable of effectively identifying multiple outliers in large datasets. Outlier analysis was conducted on individual groups, and identified outliers were excluded on a pairwise basis.

## Results

### Behavioral Results

**FIGURE 2:**
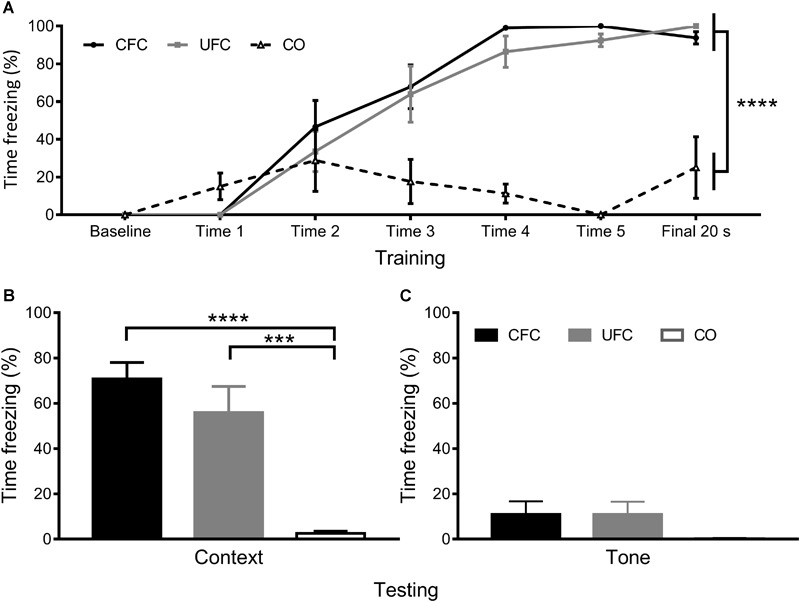
Freezing to context and tone data. Previously reported freezing to context and tone (Chaaya et al., 2019) reveal both training protocols to robustly create fear memories to context. **(A)** Analysis of fear-related freezing during conditioning reveal rats that underwent both CFC and UFC to progressively develop fear memories. At baseline, freezing behavior is 0% for all groups. By cue 1, rats in the CO control had a random (but not significant) increase in freezing, while the two fear conditioned groups still had 0% freezing. Freezing in these two fear conditioned groups began to rise by cue 2. By the final 20 second period, rats in the CFC and UFC both expressed significantly more fear-related freezing to context as compared to the CO control. **(B)** During the FMT to context provided 24 h following conditioning, rats in both the CFC and UFC expressed significantly more fear-related freezing behavior as compared to the CO control. There was no difference in fear-related freezing between the CFC and UFC group. **(C)** During the FMT to tone provided 3 days after the FMT to context, fear-related freezing was equivalent in all groups. CFC, contextual fear conditioning; UFC, unpaired fear conditioning; CO, context only; FMT, fear memory test. Asterisks denote level of statistical significance between groups ^∗^*p* ≤ 0.05; ^∗∗^*p* ≤ 0.01; ^∗∗∗^*p* ≤ 0.001; ^∗∗∗∗^*p* ≤ 0.0001.

In order to confirm that the behavioral procedures produced differences in fear to context, freezing behavioral during training and testing was quantified. The percentage of time rats displayed freezing behavior as a function of condition (CFC, UFC, or CO) and time-point (baseline minute, cue 1–5, and final minute) were quantified as presented previously (Chaaya et al., 2019). A two-way mixed design ANOVA revealed a significant interaction of freezing behavior as a function of condition and time-point (*p* < 0.0001), a significant main effect of condition (*p* < 0.0001) and a significant main effect of time-point (*p* < 0.0001). Bonferroni corrected *post hoc* tests (see Figure 2A) demonstrated a progressive acquisition of fear to context, becoming significantly different between both conditioned groups as compared to the CO control group starting from cue 4. One-way ANOVA of freezing during the FMT to context revealed significant differences between groups (*p* < 0.0001). Bonferroni corrected *post hoc* tests revealed rats that underwent CFC and UFC to exhibit significantly more freezing to context as compared to rats in the CO control group (Figure 2B). Alternatively, one-way ANOVA of freezing during the FMT to tone revealed no statistical differences to exist between groups (see Figure 2C).

### Anatomical Results

#### pCREB Expression Does Not Changed by Both CFC and UFC

**FIGURE 3:**
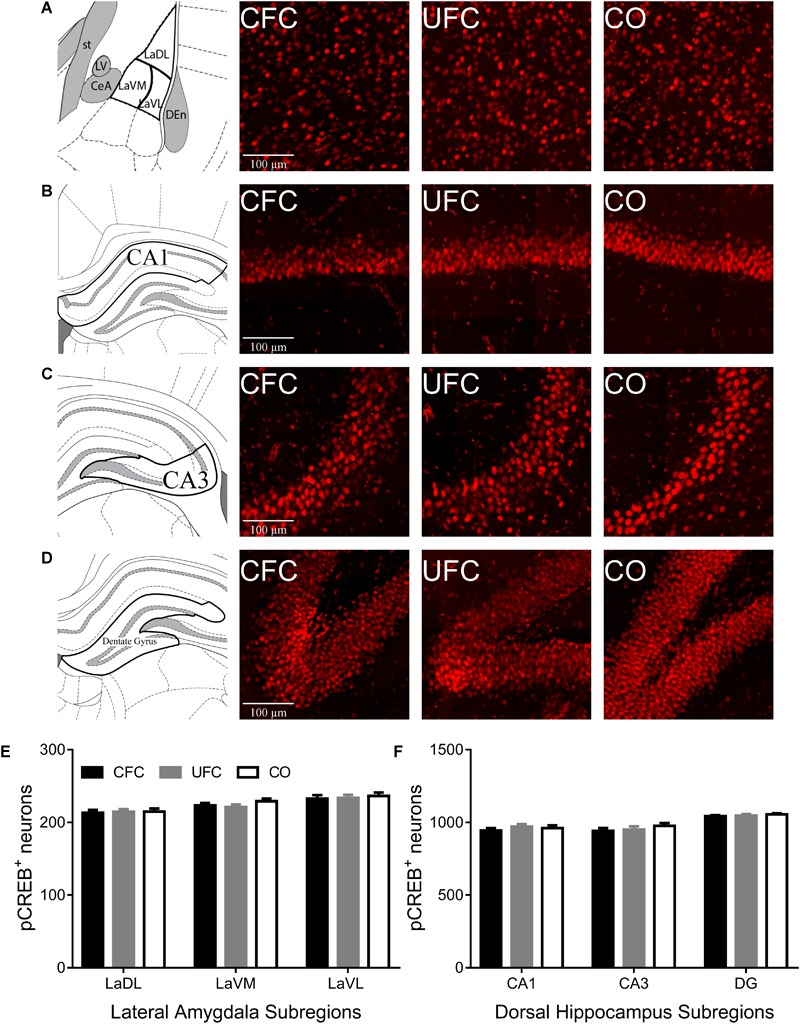
pCREB expression in LA and DH. Representative images from each behavioral sub-group of labeled pCREB in the **(A)** LA, **(B)** CA1, **(C)** CA3, and **(D)** DG subregion of the dorsal hippocampus. Evaluation of pCREB expression in LA **(E)** and its subregions and DH **(F)** and its subregions revealed no statistically significant differences as a function of conditioning. pCREB, phosphorylated cyclic-AMP response element binding; LA, lateral amygdala; DH, dorsal hippocampus; CA1, hippocampal subregion CA1; CA3, hippocampal subregion CA3; DG, dentate gyrus.

The number of neurons expressing the plasticity-related protein, pCREB, was quantified and compared in rats from the CFC, UFC and CO control groups. Analyses were specific to LA and its three subregions (LaDL, LaVM, and LaVL) as well as the DH and its three major subregions (CA1, CA3, and DG). One-way ANOVA revealed no group differences to exist (see Figure 3). No differences in pCREB number were observed as a function of conditioning in LA subregions LaDL [*F*(2,55) = 0.0431, *p* = 0.9578], LaVM [*F*(2,55) = 1.282, *p* = 0.2856], or LaVL [*F*(2,55) = 0.1709, *p* = 0.8433]. Similarly, no group differences were observed in DH subregions CA1 [*F*(2,61) = 0.5423, *p* = 0.5842], CA3 [*F*(2,60) = 0.7473, *p* = 0.4780] and DG [*F*(2,60) = 0.5430, *p* = 0.5838].

#### BDNF Expression in DG Is Increased by CFC

**FIGURE 4:**
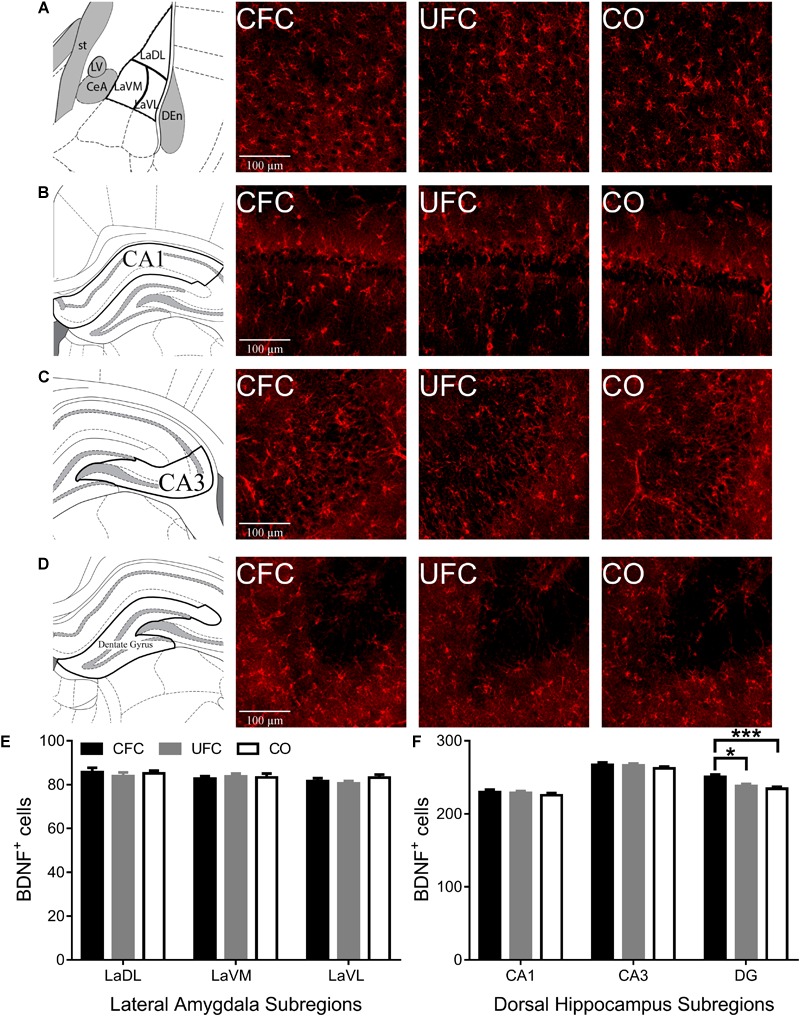
BDNF expression in LA and DH. Representative images from each behavioral sub-group of labeled BNDF in the **(A)** LA, **(B)** CA1, **(C)** CA3, and **(D)** DG subregion of the dorsal hippocampus. **(E)** Evaluation of BDNF expression in LA and its subregions revealed no statistically significant differences as a function of conditioning. **(F)** Evaluation of BDNF expression in DH revealed statistically significant differences in subregion DG as a function of condition. Rats that underwent CFC had significantly more BDNF expression as compared to both rats that underwent UFC and the CO control rats. BDNF, brain derived neurotrophic factor; LA, lateral amygdala; DH, dorsal hippocampus; CA1, hippocampal subregion CA1; CA3, hippocampal subregion CA3; DG, dentate gyrus; CFC, contextual fear conditioning; UFC, unpaired fear conditioning; CO, context only. Asterisks denote level of statistical significance between groups ^∗^*p* ≤ 0.05; ^∗∗^*p* ≤ 0.01; ^∗∗∗^*p* ≤ 0.001; ^∗∗∗∗^*p* ≤ 0.0001.

Following quantification of neurons expressing pCREB, the number of cells expressing BDNF was quantified in LA and DH, and compared between groups. One-way ANOVA of BDNF expression in LA and DH subregions CA1 and CA3 provided mostly similar results as that of pCREB expression. No differences in BDNF expression were noted in LA (see Figure 4E) subregion LaDL [*F*(2,57) = 0.3216, *p* = 0.7263], LaVM [*F*(2,56) = 0.1123, *p* = 0.8940] or LaVL [*F*(2,57) = 1.0571, *p* = 0.3542]. Furthermore, no differences in BDNF expression were noted in DH subregion (see Figure 4F) CA1 [*F*(2,56) = 0.6008, *p* = 0.5519] or CA3 [*F*(2,56) = 0.8187, *p* = 0.4462]. Contrary to pCREB data above, one-way ANOVA revealed a significant difference in BDNF expression as a function of condition in DH subregion DG [*F*(2,55) = 8.1509, *p* < 0.001]. Bonferroni correct *post hoc* tests (see Figure 4F) found rats that underwent CFC to have significantly more BDNF expression as compared to the control group. Interestingly, rats that underwent CFC also exhibited significantly more BDNF expression in DG as compared to those that underwent UFC.

#### IBA-1 Number in CA1 and DG Is Increased by CFC

**FIGURE 5:**
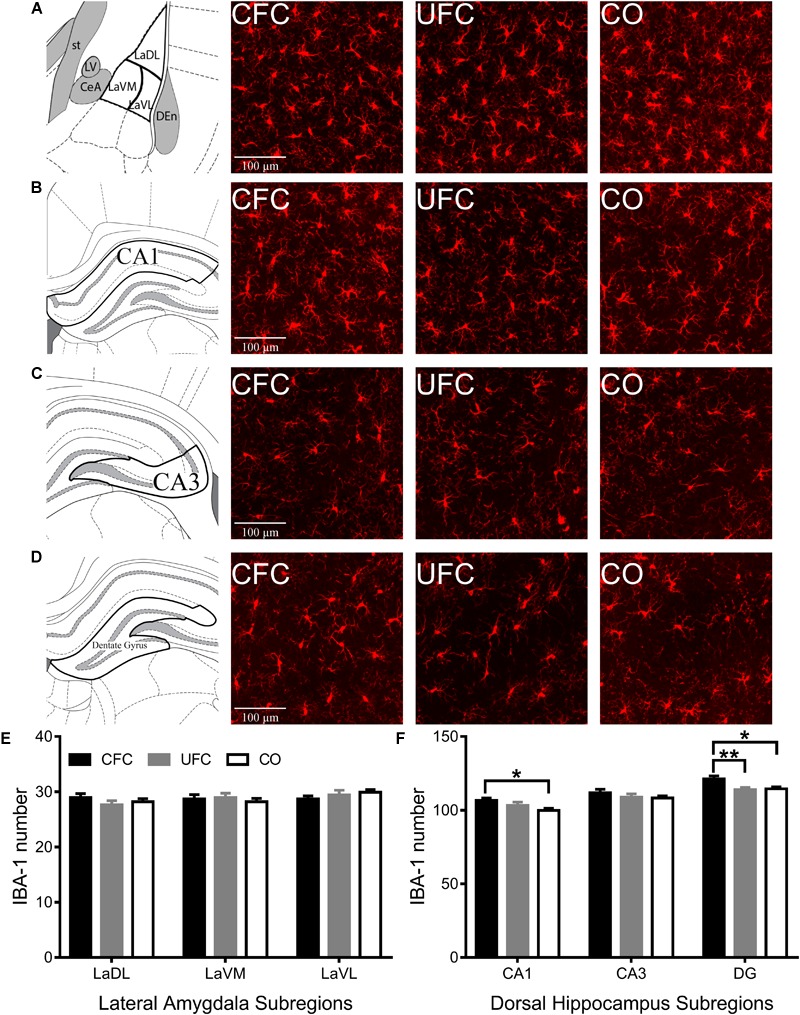
IBA-1 expression in LA and DH. Representative images from each behavioral sub-group of labeled pCREB in the **(A)** LA, **(B)** CA1, **(C)** CA3, and **(D)** DG subregion of the dorsal hippocampus. **(E)** Evaluation of IBA-1 number in LA and its subregions revealed no statistically significant differences as a function of conditioning. **(F)** Evaluation of IBA-1 number in DH revealed statistically significant differences in subregions CA1 and DG as a function of condition. Rats that underwent CFC had significantly more IBA-1 as compared to the CO control in subregions CA1 and DG. Furthermore, rats that underwent CFC had significantly more IBA-1 than rats that underwent UFC in the DG. IBA-1, ionized calcium binding adaptor molecule 1; LA, lateral amygdala; DH, dorsal hippocampus; CA1, hippocampal subregion CA1; CA3, hippocampal subregion CA3; DG, dentate gyrus; CFC, contextual fear conditioning; UFC, unpaired fear conditioning; CO, context only. Asterisks denote level of statistical significance between groups ^∗^*p* ≤ 0.05; ^∗∗^*p* ≤ 0.01; ^∗∗∗^*p* ≤ 0.001; ^∗∗∗∗^*p* ≤ 0.0001.

To determine how CFC and UFC alter microglia in LA and CA1, microglia (labeled with IBA-1) were quantified. Similar to pCREB and BDNF expression, one-way ANOVA of IBA-1 number revealed no differences in the LA (see Figure 5E) as a function of conditioning. The number of IBA-1 remained the same in subregions LaDL [*F*(2,57) = 0.9217, *p* = 0.4037], LaVM [*F*(2,58) = 0.3137, *p* = 0.7320] and LaVL [*F*(2,58) = 0.9302, *p* = 0.4003] as a function of conditioning. Alternatively, in DH (see Figure 5F), one-way ANOVA revealed differences in the number of IBA-1 in subregions CA1 [*F*(2,55) = 3.4574, *p* < 0.05] and DG [*F*(2,54) = 5.928, *p* < 0.01], but not CA3 [*F*(2,53) = 0.8517, *p* = 0.4324]. Bonferroni corrected *post hoc* tests (see Figure 5F) showed rats that underwent CFC to have significantly more IBA-1 as compared to the CO control in subregions CA1 and DG. Similar to BDNF data, IBA-1 number in DG was also significantly higher in the CFC group as compared to the UFC group in DG.

#### IBA-1 Morphology in DG Is Altered by CFC

**FIGURE 6:**
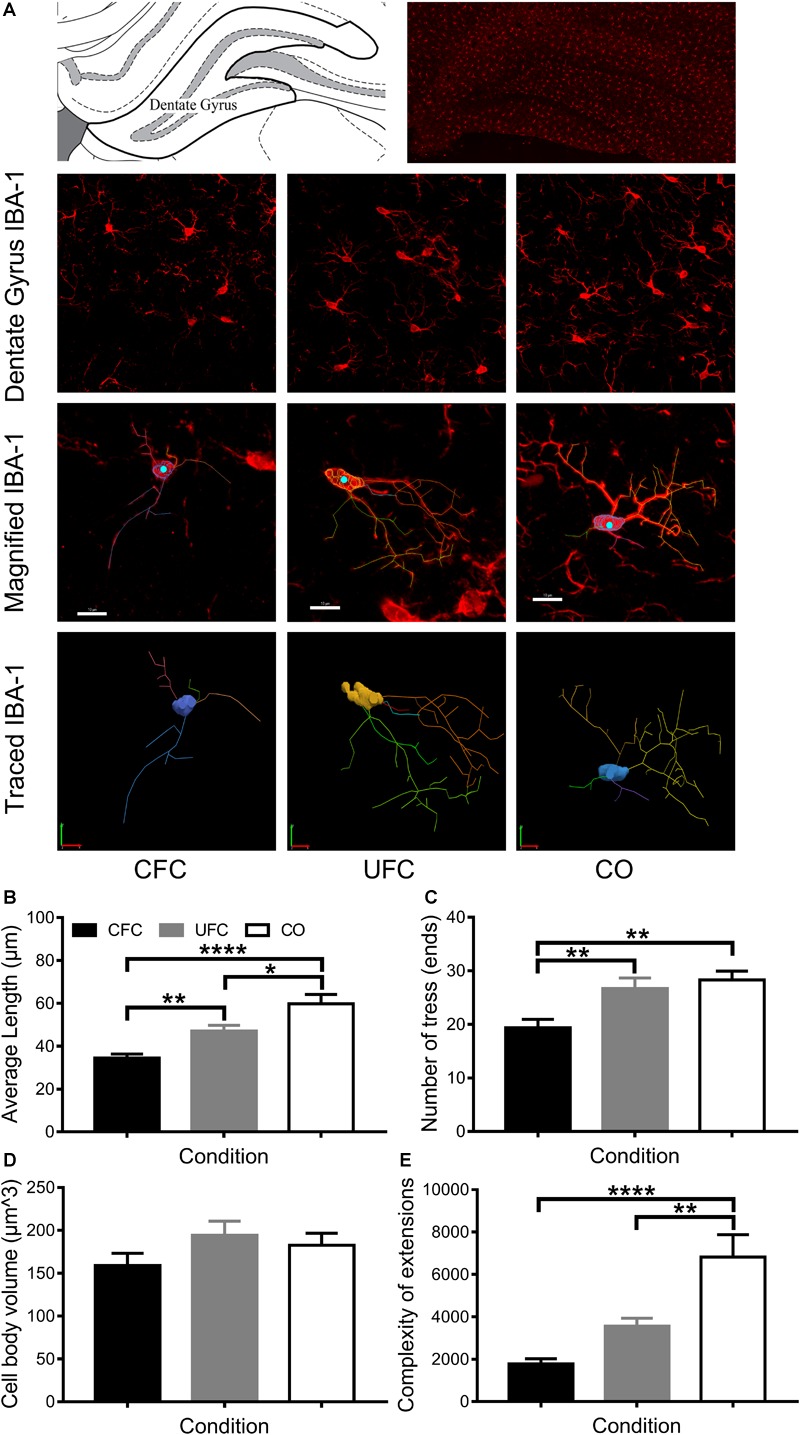
IBA-1 morphology in DG. **(A)** Representative image of DG visually demonstrating differences in IBA-1 number and morphology. The 3D projection of scanned microglia were manually traced in each behavioral group (maximum of three microglia cells per section were traced; approximately 25–30 per group). These traces provided data regarding the average length of extensions, the number of trees/endings, the cell body volume and the complexity of the extensions. **(B)** Rats that underwent CFC were found to have the shortest average length of extensions. Their extensions were significantly shorter than those in the CO control group. Rats that underwent UFC had significantly longer extensions than those in the CFC group, but significantly shorter extensions than those in the CO control group. **(C)** Rats that underwent CFC were found to have significantly less endings as compared to both the UFC group and the CO control group. **(D)** No difference microglia cell body volume was identified as a function of fear conditioning. **(E)** Analysis of the complexity of extensions (providing a measure of microglia ramification state) revealed the CFC and UFC group to both have significantly less complex extensions than the CO control group. This indicates that only the CO group had ramified or resting microglia. **(E)** Max projection of IBA-1 microglia with tracing overlaid (top) and the tracing alone (bottom). DG, dentate gyrus; CFC, contextual fear conditioning; UFC, unpaired fear conditioning; CO, context only; IBA-1, ionized calcium binding adaptor molecule 1. Asterisks denote level of statistical significance between groups ^∗^*p* ≤ 0.05; ^∗∗^*p* ≤ 0.01; ^∗∗∗^*p* ≤ 0.001; ^∗∗∗∗^*p* ≤ 0.0001. Scale bar = 10 μm.

Due to consistent differences in BDNF expression and IBA-1 number between groups (CFC versus CO control) following conditioning, IBA-1 was traced in DG (see Figure 6A). Four measures were obtained from traced IBA-1: average length of extensions, number of ends, cell body volume and complexity (as a measure of IBA-1 ramification or activation state). One-way ANOVA revealed significant differences in IBA-1 morphology in average length of extensions [*F*(2,76) = 18.3192, *p* < 0.0001], number of ends [*F*(2,80) = 8.1239, *p* < 0.001], complexity [*F*(2,70) = 17.2069, *p* < 0.0001], but not cell body volume [*F*(2,80) = 1.493, *p* = 0.2308] (see Figure 6B–E). Bonferroni corrected *post hoc* tests (see Figure 6B–E) revealed rats in the CFC group to have significantly smaller extensions than both the UFC group and CO control group. Interestingly, despite no difference in IBA-1 number between UFC and CO controls, rodents in the UFC had significantly smaller extensions than the CO control. Bonferroni corrected *post hoc* tests revealed rats in the CFC group to have significantly less ends than those in the UFC and CO control group. No differences were observed between the UFC group and CO control group. Finally, statistical analyses on complexity data revealed rats in the CFC and UFC group to have less complex (or less ramified), IBA-1 as compared to the CO control group.

## Discussion

This study investigated the involvement of amygdala and hippocampus following the formation of contextual fear memories created via separate protocols. The primary difference between these two protocols was the amount of time spent in the fear conditioning context (660 s for rats in the CFC group versus 880 s for rats in the UFC group), and importantly, the inclusion of five non-reinforced auditory tones for rats in the UFC group. Expression of pCREB and BDNF, along with the number of IBA-1 were quantified and compared in these two groups to a CO control group that explored the same context, but with no stimuli provided. Behavioral results (previously reported in Chaaya et al., 2019) demonstrate successful acquisition of fear to context, but not to tone, in both conditioned groups. Despite these behavioral changes, investigations of LA pCREB, BDNF and IBA-1 did not reveal any changes following either CFC or UFC. These data suggest the markers examined here may not be capable of identifying alterations in the LA that exist following these forms of conditioning protocols. Contrary to this, differences in BDNF and IBA-1 number were present in hippocampus following CFC, but not UFC. Specifically, an increase in the expression of BDNF in hippocampal subregion DG, and IBA-1 in hippocampal subregions CA1 and DG was present. Interestingly, DG-specific BDNF and IBA-1 were significantly higher following CFC as compared to both the CO control group, and the UFC group. These data suggest that contextual fear memories created with standard CFC protocols have a time-dependent reliance (90 min following learning) upon the DG. However, when contextual fear memories are altered with non-reinforced auditory tones, dependency for the DG appears to be lost – perhaps being dependent upon other brain regions, or upon the DH (as a whole) at different time-points.

### Lateral Amygdala

Contextual fear memories created with either the standard CFC protocol or the altered UFC protocol do not appear to rely upon pCREB, BDNF, or IBA-1 microglia in LA when measured 90 min following conditioning. These data appear to contradict previous reports (Impey et al., 1998; Hall et al., 2000; Malkani and Rosen, 2000; Hall et al., 2001; Perez-Villalba et al., 2008; Barot et al., 2009; Wilson and Murphy, 2009; Fanselow, 2010; Trogrlic et al., 2011; Pignataro et al., 2013; Zelikowsky et al., 2014; Chaaya et al., 2018). Furthermore, these results are in direct contrast with our previous investigations, demonstrating wide scale BLC activation following UFC, and specific LaDL activation following CFC (Chaaya et al., 2019). The particular proteins and cells explored here may explain this discrepancy. For example, early research could not identify any changes in LA BDNF expression following CFC (Hall et al., 2000). Further research found amygdala BDNF to increase as a function of cue fear conditioning, but not as a function of contextual fear conditioning (Rattiner et al., 2004). Similar to this, investigations into microglia following fear conditioning appears to be limited, with only recent research beginning to examine its functional role in psychiatric disorders (Dwyer and Ross, 2016). For example, a recent study demonstrated the requirement for microglia activation in chemically induced fear (Vollmer et al., 2016), with numerous other investigations showing microglia to respond to stressful stimuli (Nair and Bonneau, 2006; Frank et al., 2007; Tynan et al., 2010; Calcia et al., 2016). However, no research to our knowledge has specifically examined the alterations in microglia number and morphology that occur following fear conditioning protocols. The data here, therefore, provides evidence that microglia number in LA remains stable following contextual fear conditioning. Additionally, despite many documented cases of altered activity and protein expression in LA following CFC (see recent review Chaaya et al., 2018), data reported here confirm that BDNF expression in LA is unchanged.

Contrary to BDNF and IBA-1 data, pCREB expression in amygdala following CFC has been examined. Early research on CRE-lac Z transgenic mice investigated CREB (expressed as β-galactosidase in these mice) expression following contextual, unpaired and cued fear conditioning (Impey et al., 1998). They noted an increase in amygdala CREB as a function of both CFC and UFC (Impey et al., 1998). More recent research conducted in two phases demonstrated that (1) oral administration of DMP696, a corticotropin-releasing factor 1 antagonist significantly reduced BLC pCREB expression and subsequent contextual fear expression, and (2) bilateral microinjections of DMP696 in BLC significantly reduce fear-related freezing to context (Hubbard et al., 2007). Interestingly, DMP696 had no effect on CeA pCREB expression, and microinjections of the antagonist into the CeA did not affect fear-related freezing to context (Hubbard et al., 2007). This suggests the deficits in fear-related freezing to context occurred due to disturbances in contextual fear learning, as opposed to fear-expression. Numerous other investigations have also confirmed a role for amygdala pCREB expression in CFC (Stanciu et al., 2001; Mamiya et al., 2009; Hagewoud et al., 2011). Considering these studies, the results reported here are unexpected, and may be attributed to methodological confounds. These are explained in detail below.

### Dorsal Hippocampus

The data reported here suggest that CFC, but not UFC, rely upon BDNF and IBA-1 microglia in the DG subregion of the hippocampus, when measured 90 min following conditioning. Following CFC, BDNF expression was significantly increased in DG, while IBA-1 number was significantly increased in DG and CA1 as compared to the CO control. However, similar to above, no difference in pCREB expression was present as a function of conditioning. Once again, this pCREB data is contradictory to previous reports. On numerous occasions, hippocampal pCREB has been found to be essential to CFC (Impey et al., 1998; Stanciu et al., 2001; Kudo et al., 2004; Trifilieff et al., 2006; Mamiya et al., 2009; Hagewoud et al., 2011). Nevertheless, the increase in BDNF expression is expected. Several studies have found a functional role for hippocampal BDNF following CFC (Hall et al., 2000; Barrientos et al., 2004; Liu et al., 2004; Bekinschtein et al., 2007; Lubin et al., 2008; Takei et al., 2011; Mizuno et al., 2012). However, the majority of this research found CA1 BDNF to be essential for CFC (Hall et al., 2000; Lee et al., 2004; Bekinschtein et al., 2007; Lubin et al., 2008). Our recent review also concluded that the DH subregions most involved in CFC appear to be the CA1 and CA3, but not the DG (Chaaya et al., 2018). Nevertheless, some evidence has found increases in BDNF expression in the DG (Barrientos et al., 2004), with many other studies showing the requirement for BDNF in the DH as a whole (Liu et al., 2004; Takei et al., 2011; Mizuno et al., 2012). The data here, therefore, suggest that DG BDNF is involved in contextual fear memory consolidation.

This work showed a significant increase in the number of IBA-1 in hippocampal subregions CA1 and DG. Examination of microglia morphological states within the DG revealed altered branching and processes. As compared to CO controls, rats that underwent CFC had significantly shorter extensions, with fewer ends and less complex extensions, suggesting a clear morphological response to this fear conditioning protocol. An increase in the number of microglia (via the blood system or proliferation) typically occurs due to central nervous system response to harmful stimuli (Kettenmann et al., 2011; Calcia et al., 2016). Additionally, microglia alter morphology in response to harmful stimuli (Kettenmann et al., 2011; Calcia et al., 2016; Dwyer and Ross, 2016). During resting phase, without harmful stimuli, microglia are ramified; when responding to harmful stimuli, microglia become amoeboid (Calcia et al., 2016). When ramified, or “resting,” microglia have long, thin extensions with many processes that search for signals of insult (Kettenmann et al., 2011; Walker et al., 2014; Dwyer and Ross, 2016). When amoeboid, microglia extensions retract, cell bodies enlarge, and they respond to insult by release of proinflammatory and immunoregulatory factors and compounds (Kettenmann et al., 2011; Walker et al., 2014; Dwyer and Ross, 2016). One such factor that can be released by microglia in response to injury is BDNF (Ferrini and De Koninck, 2013; Pósfai et al., 2018). Microglia in the DG following CFC in the current study were found to represent the typical morphological state of those responding to insult. Not only did they represent the morphological structure of an amoeboid microglia, it is possible that they may have influenced the increase in BDNF. Microglia are known to control and modulate neuronal plasticity via contact-dependent mechanisms and via the release of BDNF (Wohleb, 2016). Further research is, therefore, required to confirm the direct relationship of microglia and the plasticity-related protein, BDNF in contextual fear. Provided a link exists, results from this study may have clinical benefit. Briefly, neuronal activity and plasticity in hippocampus alter continuously from a number of emotionally significant and insignificant stimuli (Bostock et al., 1991; Anderson and Jeffery, 2003; Jeffery et al., 2003; Smith and Mizumori, 2006). However, microglia are documented to alter only following the introduction of harmful stimuli in the brain (Kettenmann et al., 2011; Walker et al., 2014; Pósfai et al., 2018). In this case, the harmful stimulus was a fearful memory. If microglia are found to directly alter neuronal plasticity, a novel, and side-effect free (or side-effect reduced) method for reducing the emotional and neuroanatomical consequences of pathological fear can be developed. The inhibition of microglia may stop the production of plasticity that leads to long-term and pathological fear memories, while not affecting other plasticity and activity required for normal brain function.

Both BDNF and IBA-1 number in DG were significantly higher in the CFC group as compared to the UFC group. The UFC group has typically been used as a control to cued fear conditioning groups (McKernan and Shinnick-Gallagher, 1997; Rogan et al., 1997; Majak and Pitkänen, 2003; Radley et al., 2006; Bergstrom et al., 2011, 2012). This is because the UFC do not have overlapping cue and foot-shock, and therefore no associative memory is formed (Romanski et al., 1993; LeDoux, 2003). Nevertheless, during CFC, the “context” becomes paired with the foot-shock (Phillips and LeDoux, 1994; Desmedt et al., 1998; Calandreau et al., 2005; Calandreau et al., 2006; Trifilieff et al., 2006, 2007). While this context is altered during the UFC protocol, it still gets paired with the foot-shock. We previously showed the UFC protocol to result in wide-spread BLC activation, whereas the CFC protocol only resulted in specific LaDL activation (Chaaya et al., 2019). Contrastingly, despite equivalent levels of fear to context, the UFC protocol here resulted in significantly less BDNF and IBA-1 number in DG as compared to the CFC protocol. We hypothesize that this occurred as a result of the auditory tones altering the contextual fear memories. Trace fear conditioning is a behavioral paradigm similar to the UFC protocol; with the only difference being that the auditory tones are presented before a foot-shock (the period in between the tone and foot-shock represent the trace period) in a consistent and ordered manner (e.g., 10 s before each foot-shock) (Rogers et al., 2006). While the DH is well-documented to be essential to CFC (reviewed in Chaaya et al., 2018), some research has shown that it may be less involved in trace fear conditioning (Rogers et al., 2006). Nevertheless, the majority of research has reported the DH to be essential to trace fear conditioning (Quinn et al., 2005; Pierson et al., 2015; Reichelt et al., 2015). This suggests the difference between the UFC protocol and trace fear conditioning protocol may drastically change the need for DH. Contrary to BDNF and IBA-1 number data, traced microglia in DG did appear to have some morphological alterations (significant decrease in length of extensions and complexity of extensions as compared to CO controls) following UFC. Given that both protocols successfully produce contextual fear memories, further research is required to fully delineate DH involvement following CFC and UFC.

### Technical Considerations and Future Direction

Evaluation of BDNF and IBA-1, but not pCREB revealed a functional role for DG following CFC. The stability of pCREB expression in hippocampus, and also amygdala, was in direct contrast to many previous investigations (Impey et al., 1998; Stanciu et al., 2001; Kudo et al., 2004; Trifilieff et al., 2006; Mamiya et al., 2009; Hagewoud et al., 2011). Methodological considerations may explain these results. First, no previous research identifying a role for pCREB found a difference exactly 90 min post-learning. Various proteins and IEG’s have a peak expression time following learning (Morgan and Curran, 1991; Schafe et al., 2000; Ramírez-Amaya et al., 2005; Lonergan et al., 2010; Ivashkina et al., 2016). Anatomical differences following conditioning or learning cannot be identified if animals are sacrificed before or after this time-point (Schafe et al., 2000). Some evidence has found anatomical manipulations (salt loading, which is shown to activate neuronal plasticity related immediate early genes) to increase pCREB 90 min later (Shiromani et al., 1995). However, studies of fear conditioning mostly evaluate, and find differences in, pCREB expression 60 and 120 min following conditioning (Stanciu et al., 2001; Lee et al., 2004; Izumi et al., 2008). Therefore, while the current study found no role for pCREB in CFC or UFC, further research is required to investigate expression levels at various time-points following learning.

The further caveat of the current project is the small differences in actual number of BDNF (mean difference of 16.17 between CFC and CO control) and IBA-1 (mean difference of 7.167) number. The small standard errors make these differences statistically significant. Nevertheless, the change in number may limit the clinical significance of this study. Targeted pharmacological inhibitors can produce large-scale alterations in protein expression (see pCREB study Hubbard et al., 2007). While large-scale alterations may be beneficial to the treatment of fear-related disorders, it may have numerous confounding behavioral consequences. While this particular caveat exists in all neuroscientific research, it appears to be exaggerated by the atypically small number in differences reported here. Nevertheless, identification of these differences may be essential to understanding how the healthy brain can become pathological. For example, the small increase in BDNF and IBA-1 number lead to more in-depth analyses into microglia morphology, which were found to be have large and clear alterations.

Research has shown that microglia can release BDNF once activated (Ferrini and De Koninck, 2013; Pósfai et al., 2018). In this study, evaluation of the DG revealed an increase in microglia number, a change in microglia morphology, as well as an increase in BDNF expressing cells following CFC. The corresponding changes in microglia and BDNF expression suggest a causal relationship, whereby microglia released BDNF. However, a particular limitation of the current study is an inability to show this link. Future research, therefore, is required to evaluate the relationship between BDNF and microglia. Specifically, studies involving other neuroscientific approaches, such as co-labeling of these molecules, genetically modified knock-out mice or chemogenetic inhibition of microglia or BDNF can clearly demonstrate whether a causal relationship exists. Specific investigations within the DG following CFC are required to determine how the relationship between these molecules alters and influences contextual fear memory formation.

In the current experiment, we provide some of the first insights into how Pavlovian fear conditioning protocols alter microglia morphology. However, in this study, we are unable to accurately determine what induces these changes in microglia – fear memory formation or general stress induced from foot-shocks. Some recent evidence has demonstrated fear memory formation to alter microglia in a similar manner that is reported here (Vollmer et al., 2016). In this previous study, microglia number and morphology was altered in sensory circumventricular organs (brain structures that are in contact with blood and cerebrospinal fluid) following chemically induced fear (Vollmer et al., 2016). However, a limitation of this study is that the changes in microglia may be attributed to carbon dioxide (which was utilized to evoke intense fear and panic in mice) directly changing brain circuits, as opposed to carbon dioxide causing fear, which altered microglia (Vollmer et al., 2016). Besides from this study, no prior research has examined how fear memory formation alters microglia number and morphology. Nonetheless, previous investigations have shown how stress induced by inescapable electric shocks can alter microglia in hippocampus (Frank et al., 2007). These data suggest foot-shock alone (from the current study) may be responsible for the alterations in microglia – and not fear memory formation. Unfortunately, our specific methodology does not allow us to identify the direct cause of microglia alterations. Nonetheless, an argument can be made for fear memory formation altering microglia. In this study, we show differences in microglia number and morphology between the CFC and UFC groups. Interestingly, these groups were presented with identical electric foot-shocks which produced statistically similar fear to context. The main difference between these groups is that the UFC protocol had randomly presented non-reinforced auditory tones. These data indicate that alterations to the fear conditioning procedure can alter microglia number and morphology – indicating that foot-shock presentation alone is not driving the alterations reported in this study. Despite this, further research is required to directly determine whether fear memory formation or foot-shock alone alter microglia morphology. By utilizing memory reconsolidation experimental designs (whereby fear memory is reactivated hours or days following conditioning – resulting in the brain regions required for consolidation to be reactivated), future research can explore how the reactivation of fear, and not presentation of electric foot-shock, alters microglia (Nader et al., 2000).

## Conclusion

The current study investigated how two differing contextual fear memories are represented in the rat brain. While fear to context was relatively constant, we report differences in BDNF and IBA-1 number between these two conditioned groups. Standard CFC leads to an increase in BDNF expression, IBA-1 number, while UFC did not. Furthermore, we provide some of the first data showing microglia morphology to become altered as a function of CFC and UFC. Interestingly, while BDNF expression and IBA-1 number increased only in following CFC, morphological differences were identified in both the CFC and UFC as compared to the CO control. While the change in BDNF and IBA-1 number may have limited clinical significance, the alteration in microglia morphology may be clinically significant. When active, or amoeboid, microglia can alter neuronal activity via the BDNF-TrkB pathway (Ferrini and De Koninck, 2013; Pósfai et al., 2018), and therefore targeted treatment of microglia may affect only the necessary neurons responding to fear-inducing stimuli. This suggests that targeted drug treatments aimed at inhibiting microglia activity (Greter and Merad, 2013; Cheng et al., 2015) may provide a new therapeutic tool for sufferers of fear-based disorders. Further research is required to investigate how reducing microglia activity influences fear memory consolidation and maintenance.

## Data Availability

The raw data supporting the conclusions of this manuscript will be made available by the authors, without undue reservation, to any qualified researcher.

## Ethics Statement

All behavioral procedures were approved by the University of Queensland (Ethics Approval No. 023/17) and Queensland University of Technology (QUT Approval No. 1700000295) animal ethics unit. All procedures complied with the Queensland Government Animal Research Act 2001, associated Animal Care and Protection Regulation (2002 and 2008), as well as the Australian Code for the Care of Animals for Scientific Purposes, 8th Edition (National Health and Medical Research Council, 2013) policies and regulations of animal experimentation and other ethical matters.

## Author Contributions

NC designed the study, conducted the behavioral protocols, laboratory work, imaging, and data analysis, interpreted the data, and typed/edited the manuscript. AJ assisted with behavioral protocols, laboratory work, and imaging. AB assisted with laboratory work, imaging, and editing of manuscript. KB and SA assisted with laboratory work. FC assisted with interpretation of data and editing of manuscript. ARB assisted with editing of manuscript. LJ assisted with design of study and behavioral protocols. SB assisted with design of study and interpretation of results.

## Conflict of Interest Statement

The authors declare that the research was conducted in the absence of any commercial or financial relationships that could be construed as a potential conflict of interest.
